# Public perception of genetically modified organisms (GMOs) in Montenegro: insight for sustainable biotechnology and policy development

**DOI:** 10.1080/21645698.2026.2620884

**Published:** 2026-01-23

**Authors:** Ana Velimirović, Zoran Jovović, Siniša Berjan, Hamid El Bilali, Mohammad S. Allahyari

**Affiliations:** a Biotechnical Faculty, University of Montenegro, Podgorica, Montenegro; b Faculty of Agriculture, University of East Sarajevo, Sarajevo, Bosnia and Herzegovina; c International Centre for Advanced Mediterranean Agronomic Studies – Mediterranean Agronomic Institute of Bari (CIHEAM-Bari), Valenzano (Bari), Italy; d Faculty of Economic and Management Sciences, North-West University, Mahikeng, South Africa

**Keywords:** Biotechnology, consumer behavior, GMOs, Montenegro, public perception, risk perception

## Abstract

Genetically modified organisms (GMOs) hold significant potential for enhancing agricultural sustainability, yet public acceptance remains limited. This study examined consumer perceptions of GMOs in Montenegro, where traditional agriculture coexists with emerging biotechnology. Using an online survey of 1178 respondents, attitudes toward GMOs, health and environmental risk perceptions, and media influences were analyzed. Results showed that 76% of respondents considered GMOs harmful to human health, with strong concerns regarding environmental and animal impacts. Women and respondents with higher education levels expressed higher risk awareness. K-means clustering identified three consumer groups – Highly Concerned (34.9%), Moderately Concerned (37.9%), and Critical but Uncertain (27.2%) – differing in awareness, information sources, and consumption behavior. Findings highlight the role of misinformation and low institutional trust in shaping public attitudes. Strengthening science-based communication and transparent labeling policies is essential for fostering informed decisions and supporting the integration of biotechnology into sustainable agriculture.

## 1. Introduction

The process of selective breeding, tracing back to ancient civilizations, has been pivotal in shaping the traits of plants and animals to meet human needs.^1^ With advancements in science and technology, biotechnology has allowed for the manipulation of genomes, leading to the creation of genetically modified organisms (GMOs).^2^ While commonly associated with GM plants and food production, genome editing finds applications in human medicine and bioremediation as well.^3,4^ Engineered to express desirable traits, GMOs offer numerous advantages, including increased crop yields, reduced chemical usage, enhanced nutritional content, flavor, quality, and shelf life, and the potential to address global food insecurity.^5–7^

Despite their potential, GMOs remain one of the most controversial topics in modern agriculture. Public skepticism is largely driven by misinformation, ethical concerns, and a limited understanding of genetic modification.^8,9^ Misinformation, often amplified through digital media, shapes consumer opinions, leading to misconceptions about GMO safety, environmental impact, and long-term health effects.^10,11^ Ethical and religious debates further complicate the acceptance of GMOs, contributing to a polarized discourse between scientific consensus and public perception.^12^ Consumer attitudes toward GMOs are shaped by socio-cultural factors, risk perception, and trust in regulatory institutions. According to consumer choice theory, decision-making under uncertainty is influenced by emotional and cognitive biases rather than purely scientific evidence.^13^ Risk perception theory further explains how cultural values and institutional trust contribute to regional differences in GMO acceptance.^14–16^ Studies indicate that emotional reactions and ethical considerations often outweigh objective risk assessments, leading to persistent skepticism.^17,18^

The role of digital technologies and media in shaping consumer perceptions of GMOs is another important factor. Media discourse often amplifies concerns, coupled with misinformation and the absence of comprehensive long-term studies. Public fears are commonly influenced by unverified information, further deepening mistrust.^19^ Concerns about health risks, including allergies, digestive issues, cancer, and antibiotic resistance, persist despite the extensive regulatory frameworks governing GMOs.^20,21^ Addressing these concerns through accurate and transparent communication, along with rigorous scientific evaluation, is essential in fostering informed decision-making regarding the use of GMOs in agriculture. These dynamics demonstrate that integration of consumer behavior theories and risk perception frameworks into communication strategies is essential in order to address consumer concerns effectively and promote informed decision-making.^22^

In recent decades, consumer trust in the effectiveness and safety of food control systems has significantly decreased.^23,24^ Furthermore, awareness of the environmental and health consequences of agricultural practices increased the need to better understand their effects.^25^

Existing research on consumer perceptions of GMOs reveals a complex interplay of various socio-cultural factors, risk perceptions, and trust in regulatory institutions.^26^ Studies conducted in diverse socio-economic contexts emphasize the impact of media discourse, interpersonal communication, and socio-demographic characteristics on shaping consumer attitudes toward GMOs.^27^ There is limited empirical evidence regarding Montenegrin consumers’ perceptions of GMOs and their information-seeking behaviors. With its rich agricultural heritage and evolving regulatory framework aiming to align with the European Union, it provides a unique setting to investigate these dynamics. Montenegro stands at the crossroads of two contrasting approaches; reliance on traditional agriculture and conservation, and the growing influence of modern biotechnologies. This dynamic suits the examination of how the public perceives and accepts agricultural innovations, particularly GMOs.^28–30^

This study investigates public perceptions of agricultural innovations in Montenegro, focusing on GMOs and information sources. The research aims to explore the key factors influencing consumer perceptions of GMOs, the role of socio-cultural factors, risk perceptions, and trust in regulatory institutions in shaping attitudes, and the impact of media on public perceptions. The study hypothesizes that socio-cultural factors, risk perceptions, and trust in institutions significantly affect consumer attitudes toward GMOs, while media discourse, including misinformation, plays a critical role in shaping these perceptions. By addressing these questions, the study provides insights for policymakers, scientists, and communication strategists, contributing to more effective public engagement with agricultural biotechnology in Montenegro.

## 2. Materials and Methods

The questionnaire was developed by a team of multidisciplinary researchers from the University of Montenegro. The initial set of questions was drafted based on a thorough review of existing literature on food preferences, attitudes toward genetically modified products, and consumer behavior studies.^31–33^ Key sources included peer-reviewed journal articles and previously validated surveys.^34^ The preliminary version of the questionnaire underwent pre-testing, conducted with a small scale sample of 30 volunteers. Feedback on question clarity, format, and the overall time to complete the survey was collected. The Spearman-Brown coefficient was utilized to assess reliability, yielding a coefficient of 0.833, indicating that the questionnaire reliably measures consumer attitudes toward GMOs.

As the output, the voluntary self-administered online survey was designed utilizing the online survey platform SurveyMonkey to facilitate distribution and data collection. The final survey was disseminated over a six-week period, conducted from February 24 to April 4, 2020. A non-probability convenience sampling method was employed to disseminate the survey through various online channels. These included social media platforms, e-mail newsletters, and institutional and professional networks relevant to agriculture and food safety. While effective for reaching a wider audience, this approach may have introduced selection bias, as participation was limited to individuals with internet access and interest in the topic. Consequently, the sample may overrepresent certain demographic groups, such as younger and more educated individuals. This limitation was considered when interpreting the results. The gender distribution of respondents reflects the voluntary nature of the online survey and differential response rates commonly observed in survey-based social research, where women tend to participate more frequently than men.^35,36^ All respondents were informed about the purpose of the survey, the voluntary nature of their participation, the anonymity of their responses, and the academic use of the data collected. To maintain the integrity and validity of the survey results, responses with all sections of the questionnaire filled were considered. Responses were excluded if they contained inconsistent answers across similar items or showed patterns indicative of random completion, such as identical answers to all questions. This screening resulted in a final number of 1178 used in this study, out of 3014 visits (39,08%).

The survey questionnaire encompassed a diverse range of question types, including multiple-choice questions, Likert scale questions, and open-ended questions. To ensure comprehensive data collection, the questionnaire was structured into an introduction and two sections. The first section gathered demographic data. Respondents were grouped into age categories to analyze variations in perceptions based on generational differences. Male and female respondents were included to examine gender-based attitudes toward GMOs. Education categories were captured to assess whether knowledge influenced perceptions and attitudes on GMOs. The second section, including thirteen questions related to GMOs, explored respondents’ attitudes toward conventional and genetically modified food, focusing on factors influencing their perceptions and preferences. These questions addressed concerns about the dangers of GMOs to the environment, animal health, and human health, as well as specific diseases potentially linked to GMO consumption, such as poisoning, allergies, digestive issues, autoimmune diseases, and cancer. Additional questions explored respondents’ familiarity with specific GMO plants and animals, their consumption habits, labeling awareness, and regulations regarding GMO cultivation in Montenegro and Europe. The section also examined the use of pesticides in GMO crops and the various sources from which respondents obtained information about genetically modified and healthy food, including traditional media, internet sources, social networks, personal connections, and scientific literature.

Quantitative data derived from multiple-choice and Likert scale questions were analyzed using appropriate statistical techniques within the Statistical Package for Social Sciences (SPSS26). Descriptive statistical methods (frequency and percentage) were utilized to examine the public’s perspectives on agricultural progress in Montenegro. Chi-square test was applied to assess the association between socio-demographic features and perceptions of GMO safety, as well as respondents’ sources of GMO-related information. For questions with multiple-choice options, a multiple-response analysis was conducted, which included percentages of responses and cases.

\Cluster analysis was employed to organize data such that objects within the same cluster exhibiting greater similarity to one another than to those in different clusters.^37^ K-means clustering method was utilized for this purpose. The algorithm operates by dividing a given dataset into a predefined number of clusters (k clusters) simply and systematically. In this approach, the clusters are nonhierarchical, mutually exclusive, and ensure that each cluster contains at least one item. For a dataset comprising n observations (X1, X2, … , Xn), K-means clustering seeks to partition these observations into k (≤n) sets, i.e., S = {S1, S2, … , Sk}, to minimize the within-cluster sum of squares. In other words, its objective is to find:

arg  mins∑i=1k∑x∈S||x−μi||

where: µi is the mean of points in Si. K-means clustering allows the determination of the appropriate cluster for all categories of consumers, and regarding attribute clusters, they provide a proper description of the group.^38^

## 3. Results

### 3.1. Demographic Data

The majority of respondents were female, comprising 60.8% of the total sample (Table 1). Across different age groups, the distribution of respondents was relatively evenly spread, with the highest proportion falling within the 35–44 age range (30.1%). The youngest age group (18–24) and the second youngest (25–34) also had substantial representation, comprising 23.3% and 27.8% of the sample, respectively. Regarding education level, the majority of respondents held a university degree, accounting for 57.9% of the sample, followed by the high school education level (29.1%), Master or PhD (11.4%). The lowest percentage held respondents with elementary or no formal education (1% and 0.6% respectively). In terms of occupation, most respondents reported either full-time or part-time employment, making up 70.4% of the sample. Students constituted the second largest occupational group at 16.0%, followed by unemployed individuals at 11.8%. Retired respondents comprised the smallest proportion, making up only 1.8% of the sample.

**Table 1.** Respondents’ characteristics (*n* = 1178).

Answer choices	Responses	Percentage (%)
**Gender**		
Male	462	39.2
Female	716	60.8
**Age group (year)**		
18–24	275	23.3
25–34	328	27.8
35–44	355	30.1
45–54	166	14.1
55 and more	54	4.6
**Education level**		
Without formal education	7	0.6
Elementary school	12	1.0
High school	343	29.1
University	682	57.9
Master or PhD	134	11.4
**Occupation**		
Full-time or part-time job	829	70.4
Student	189	16.0
Unemployed	139	11.8
Retired	21	1.8

### 3.2. Safety of GMOs

The respondents’ perceptions of the potential risks of GMOs for the environment, animal health, and human health were divided into three categories: “Yes,” “No,” and “I don’t know” (Table 2). The results show that a majority of respondents (69.2%) believe that GMOs pose risks to the environment, while a smaller percentage (12%) believe the opposite. Additionally, a significant proportion of respondents (18.8%) expressed uncertainty about the environmental risks associated with GMOs. Similarly, a large majority of respondents (72.3%) perceive GMOs as harmful to animal health, while a smaller percentage (10.8%) holds a different view. A notable number of respondents (16.9%) expressed uncertainty about the potential risks of GMOs to animal health. Regarding human health, the majority of respondents (76.9%) believe that GMOs pose a danger, while a minority (8.1%) disagrees. A considerable number of respondents (14.9%) expressed uncertainty about the potential risks of GMOs to human health.

**Table 2.** Respondents’ risk perceptions associated with genetically modified organisms (GMOs) and interaction with socio-demographic features.

Safety GMOs	Percentage			Chi-square test			
	Yes	No	I don’t know				
				Gender	Age	Education	Occupation
Are genetically modified organisms dangerous for the environment?	69.2	12.1	18.8	12.33**	25.77**	11.95	25.85**
Are genetically modified organisms dangerous for animal health?	72.3	10.8	16.9	3.31	25.54**	21.86**	25.39**
Are genetically modified organisms dangerous to human health?	76.9	8.1	14.9	14.53**	13.49	26.33**	20.97**

**p<.01.

The chi-square test results revealed a statistically significant interaction effect at the 1% level of significance between respondents’ gender and their perspectives on the risks of GMOs on the environment, animal and human health. Notably, women were significantly more inclined than men to believe that GMO products pose risks to the environment and human health. Additional analysis by age group indicated that the 35–44 age group demonstrated a higher likelihood than other age groups to perceive GMO products as posing risks to the environment and animal health. The analysis revealed a significant interaction effect between respondents’ level of education and their perception of the risks associated with GMO products. Individuals with a university education were more inclined than those with other education levels to acknowledge these risks to both human and animal health. Lastly, individuals with part-time and semi-time jobs exhibited a greater tendency than other occupational groups to recognize the risks associated with GMO products on the environment, as well as human and animal health (Table 2).

### 3.3. Health Hazards of GMOs

The health issues related to genetically modified organisms (GMOs), as presented in Table 3, include poisoning, allergies, digestive problems, autoimmune diseases, cancer, and other unspecified health concerns. A relatively small portion of respondents (22.6%) attributed poisoning to GMOs. It is noteworthy that allergies emerged as the most frequently mentioned health issue associated with GMOs, with 552 respondents expressing this concern (49.8%). Additionally, a significant number of respondents linked GMOs to digestive problems (32.5%) and autoimmune diseases (35.3%). The highest number of respondents (555) attributed cancer to GMOs, accounting for 50% of the sample. A smaller percentage of respondents (2.9%) mentioned unspecified health issues as being caused by GMOs.

**Table 3.** Perceived health issues associated with GMOs.

Health hazards	Responses		Percent of cases
	n	%	
Poisoning	251	11.31	22.6
Allergies	552	24.88	49.8
Digestion	360	11.2	32.5
Autoimmune diseases	392	16.23	35.3
Cancer	555	25.02	50.0
Others	108	4.87	2.9
Total	3221	100	193.4

### 3.4. Knowledge About GM Plants and Animals

**Figure 1.**
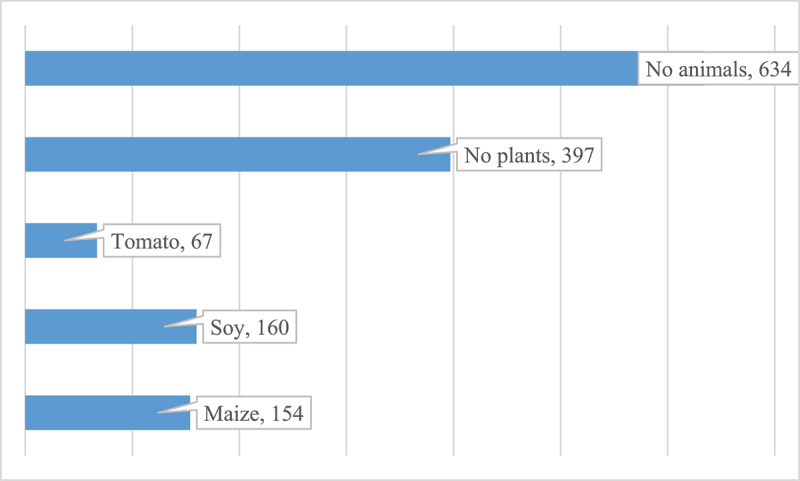
Familiarity of respondents with GM plants and animals. Source: authors.

In terms of respondents’ familiarity with GM plants and animals, the majority (634 responses, 54%) indicated that they were unaware of the existence of GM animals (refer to Figure 1). Similarly, “No” was selected 397 times (34%), indicating that a significant portion of respondents claimed to have no knowledge of GM plants. Among the responses, GM maize was mentioned 154 times, indicating that a subset of respondents is aware of maize being genetically modified. Additionally, soy crops were mentioned 160 times, while tomatoes were mentioned 67 times, making them the most frequently cited genetically modified plants. Other notable responses included wheat, potatoes, pineapples, and apples, along with mentions of sheep and lobsters (data not shown).

### 3.5. GMO Consumption and Labeling

**Figure 2.**
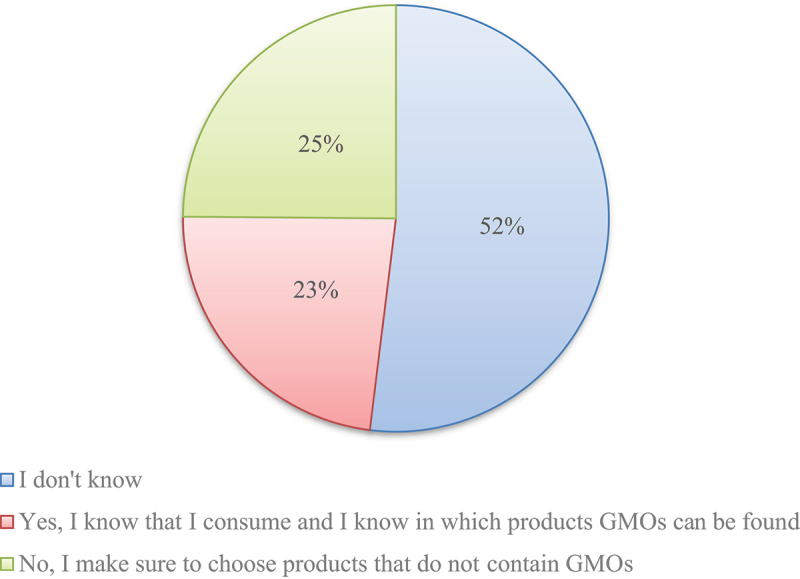
Consumer responses to GMO consumption awareness and behaviour. Source: authors.

Over half of the respondents (612, 52%) expressed uncertainty regarding their consumption of GMOs (Figure 2). A significant number of participants (271, 23%) claimed awareness of consuming GMOs and being knowledgeable about the products that contain them. A quarter of respondents (295, 25%) reported actively opting for products that are free from GMOs.

**Figure 3.**
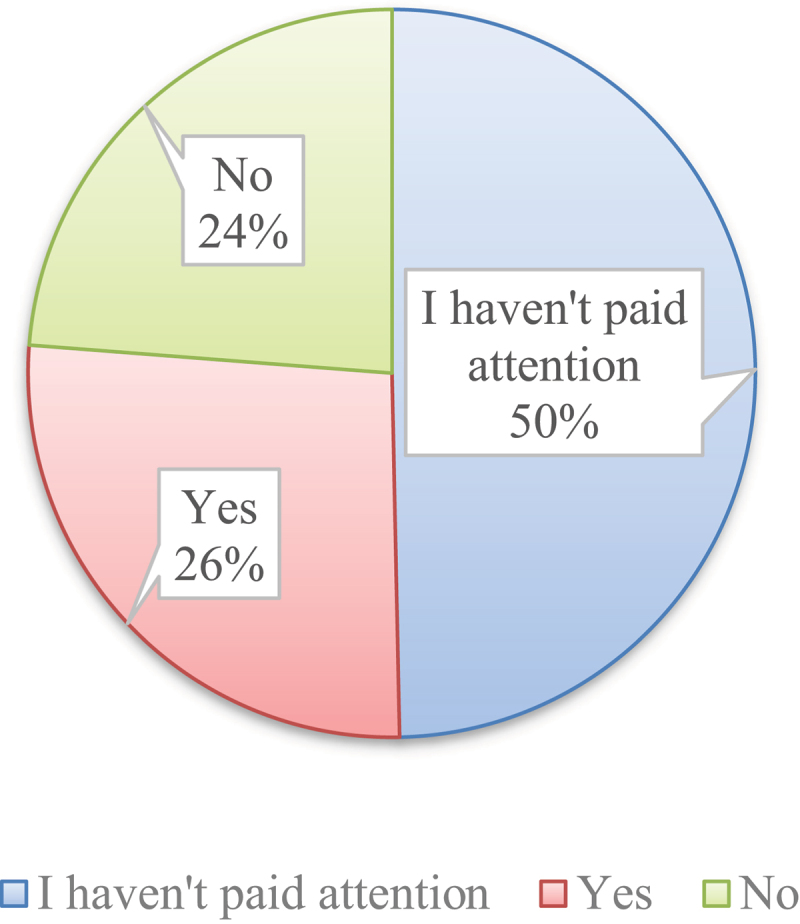
Awareness of GMO food labelling. Source: authors.

Half of the respondents (585) stated that they have not paid attention to whether products are labeled as GMOs (Figure 3). A significant portion, accounting for 26% of respondents (313), answered in the affirmative, while a smaller proportion of 24% (280) answered in the negative.

### 3.6. GM Crop Production in Montenegro and the European Union

Table 4 shows the survey results on the planting of GMOs in Montenegro and the European Union (EU). A large number of respondents (759) expressed uncertainty about whether GMOs are permitted to be planted in Montenegro. In contrast, a smaller number of respondents (190, 16%) confirmed that GMO planting is allowed in Montenegro, while a slightly higher number (229, 19%) indicated that it is not allowed. A significant portion of respondents (614, 52%) were unsure about the permissions on planting GMOs in the EU. A higher number of respondents (420, 36%) believed that GMO planting is allowed in the EU, while a lower number (144, 12%) believed it is not allowed.

**Table 4.** Perception of GMO planting regulations in Montenegro and the European Union.

	Is it allowed to plant GMOs in Montenegro?	Is it allowed to plant GMOs in the EU?
Yes	190 (16%)	420 (36%)
No	229 (19%)	144 (12%)
I don’t know	759 (65%)	614 (52%)

### 3.7. Use of Pesticides in GMOs

**Figure 4.**
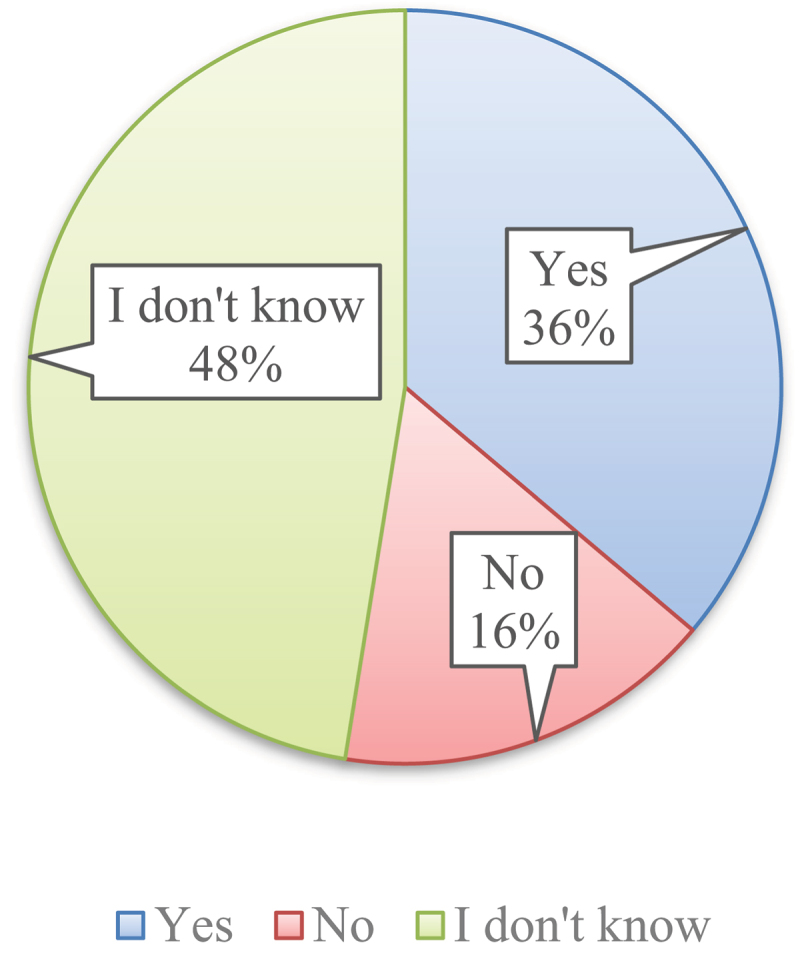
Perceptions of pesticide usage in GMO crops. Source: authors.

According to Figure 4, in response to the survey question regarding the usage of pesticides in GMO crops, the respondents’ answers were as follows: 48% (559 responses) expressed uncertainty, 36% (426 responses) answered affirmatively, and 16% (193 responses) answered negatively.

### 3.8. Sources of Information About GMOs

The majority of respondents, constituting 70.7% (Table 5), acquire information about healthy and safe food through the Internet. Approximately one-third of the participants rely on conventional media platforms such as television, radio, or newspapers, while a smaller proportion resorts to scientific literature (14.5%) for answers. The practice of obtaining information about these food categories via social networks or through personal connections with friends, neighbors, and relatives is less prevalent.

**Table 5.** Information sources of the survey participants for obtaining information.

Information sources	Responses		Percent of cases
	n	%	
TV, radio, newspaper	373	20.3	31.7
Internet	833	45.3	70.7
Friends, relatives, neighbours	159	8.6	13.5
Social networks	207	11.3	17.6
Scientific literature	267	14.5	2.7
Total	1839	100	156.1

### 3.9. Respondent Segmentation Based on Cluster Analysis

**Figure 5.**
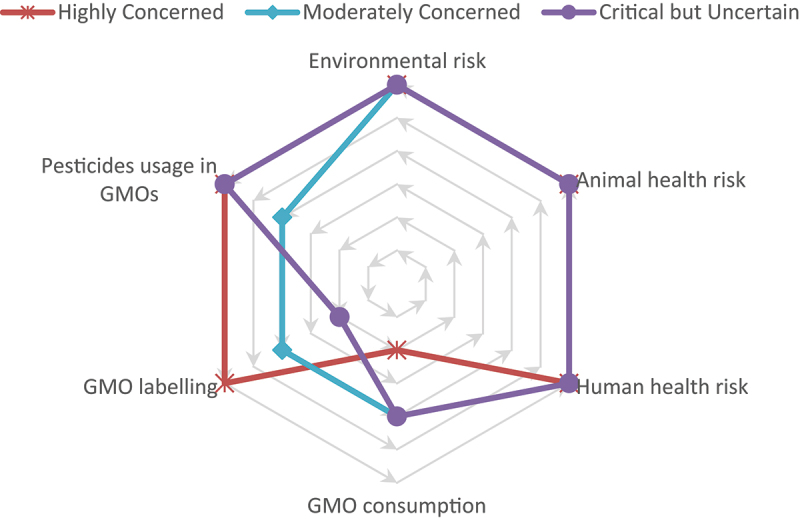
Respondent segmentation based on GMO risk perception. The figure shows three clusters identified by K-means analysis (Highly Concerned, Moderately Concerned, and critical but Uncertain) across six dimensions of perceived risk and attitudes toward GMOs.

Using K-means clustering, respondents were segmented into three distinct groups based on their perceptions of environmental, animal, and human health dangers, awareness of GMO consumption and labeling, and perceptions of pesticide usage in GMOs. After iterative testing, three clusters yielded the most meaningful results, as shown in Figure 5. Cluster 1, “Highly Concerned” (34.9%), include respondents not consuming GMOs, believing that GMOs are properly labeled, and perceiving GMOs as contributing to increased pesticide use. Their views on the environmental, animal, and human health risks associated with GMOs are notably negative. Cluster 2, “Moderately Concerned,” representing 37.9% of the respondents, shares similar concerns about the environmental, animal, and human health risks of GMOs. This group appears indifferent to issues of GMO consumption, labeling and pesticide use. Cluster 3, “Critical but Uncertain,” at 27.2%, also shares concerns about the risks associated with GMOs, especially regarding environmental, animal, and human health. However, this group is uncertain about GMO consumption and strongly believes that GMOs are not labeled. Their belief that GMOs require more pesticide use highlights a critical stance on agricultural practices, though they do not fully reject GMO products.

## 4. Discussion

Despite the widespread presence of GMOs in global food production, public knowledge remains limited, leading to persistent skepticism. This uncertainty arises from the complexity of biotechnology, which many consumers struggle to understand, and from polarized debates fueled by media narratives^39–42^. Our study confirms that health-related fears, including concerns about allergies, digestive issues, autoimmune diseases, and cancer, dominate public rejection of GMOs. Similar concerns have been observed in Lithuania^43^ and Turkey^44^, where consumers frequently perceive GMOs as health hazards. However, variations in risk perception among different regions, suggest that sociocultural factors and media exposure significantly shape public attitudes, reinforcing the risk perception theory that emotional and cognitive biases often outweigh scientific evidence^45,46^. Therefore, interactive, science-based dialogue that addresses public concerns may be more effective compared to simple information dissemination.

Our study reveals that while GMOs are associated with a reduced agricultural footprint, public concerns about their environmental impact persist, as reported in other studies^47,48^. Despite rigorous safety evaluations, concerns about the environmental, animal, and human health effects of GMOs remain prevalent, as also noted by De Vos and Swanenburg^49^. Extensive international risk assessment studies, based on rigorous pre-market evaluation, post-market monitoring, and regulatory oversight, consistently indicate that authorized GM crops are as safe as their conventional counterparts for human, animal health, and environmental health. In contrast, such comprehensive assessments were never required for conventional breeding methods that are considered safe^50–52^. In Montenegro, over a third of respondents expressed heightened concerns, which is consistent with global trends where widespread skepticism about GMOs is often driven by misinformation and cultural beliefs^53^. Similarly, the majority of misinformation related to GMOs was categorized as negative^54^. Our study also confirmed existing trends in public opinion, where health-related anxieties represent a primary driver of GMO skepticism^55^. These results challenge the deficit model of science communication, which assumes that more knowledge alone leads to greater acceptance. This aligns with the consumer behavior theory and risk perception frameworks, which emphasize the influence of cognitive and emotional biases on decision-making under uncertainty^13,17^. Therefore, communication strategies must go beyond simply disseminating factual information and instead address underlying fears and cultural values.

The findings regarding the familiarity of respondents with GMO plants and animals show a notable lack of awareness and understanding among the surveyed population. The majority of respondents (54%) indicated that they do not know about the existence of genetically modified animals, while a significant portion (34%) claimed not to know of any genetically modified plants. Similar results have been reported in previous studies. Jurkiewicz et al.^56^ found that a considerable proportion of consumers lacked awareness of GMOs, with many uncertainties about which food products are genetically modified. The recognition of GM maize and soybean aligns with their dominant presence in GMO production, whereas tomatoes are more often a recurrent public association suggests the need for accurate and transparent media coverage^12^.

A key insight from this study is the limited attention given to GMO labeling, despite clear legislation in Montenegro that mandates such labeling^57^. Over half of the respondents (52%) reported not paying attention to GMO labels, which suggests that GMO labeling may not be a significant factor in purchasing decisions. This aligns with studies showing that awareness of GMOs does not necessarily translate into active consumer behavior^58^. A substantial portion of respondents (23%) who stated that they are aware of consuming GMOs may be attributed to factors such as active information-seeking behavior. Additionally, the quarter of respondents (25%) who reported making a conscious effort to choose products that do not contain GMOs reflects a significant level of concern or preference among consumers for non-GMO options.

Uncertainty is detected among respondents regarding GMO planting regulations in both Montenegro and the EU. A large portion of participants expressed confusion about whether GMO cultivation is allowed in Montenegro, as well as within the EU. This aligns with the risk perception theory, which suggests that such uncertainty can amplify perceived risks and contribute to negative perceptions of GMOs^12^. It also reflects the importance of consumer trust in regulatory institutions. When consumers lack clear information, their trust in regulatory bodies is weakened, and they may be more likely to oppose GMOs^26^. The results support the consumer choice theory, where uncertainty often leads to emotional and cognitive biases, influencing consumer decision-making and attitudes^13,14^. Lastly, the EU’s GMO regulation framework, based on risk assessment, traceability, and labeling, reinforces the importance of transparency in ensuring the public is well-informed about GMO legislation and fostering trust in regulatory bodies^59^.

GMO crops are often engineered to be resistant to certain pesticides, allowing for increased pesticide application without harming the crop itself. Canola, soybean, cotton, and maize are widespread, herbicide-tolerant GM crops^60,61^. Nearly half of the respondents lack clarity regarding pesticide use in GM crops, demonstrating a knowledge gap about GM technologies. A lack of understanding about the specific traits of GM crops, such as pesticide resistance, can also lead to emotional and cognitive biases in decision-making, shaping public perceptions in ways that may not be entirely based on objective facts^12–14^. To address these concerns, public education must clearly differentiate between genetic modifications that reduce pesticide reliance (e.g., Bt crops) and those that involve herbicide resistance. Providing balanced, evidence-based messaging can help consumers make more informed assessment of GMOs.

The study confirms that the media is a dominant source of information on GMOs, with 70.2% of respondents relying on the internet, while traditional media (TV, radio, and newspapers) remains influential for one-third of participants. This reliance on digital platforms aligns with broader global trends in consumer information-seeking behavior^62,63^. Traditional media (television, radio, newspapers) remains influential among a third of respondents, which is consistent with existing research showing that traditional media still plays a significant role in information dissemination, particularly for older demographics^64^. The reliance on online and traditional media, where concerns and misinformation about GMOs are amplified, can heighten risk perceptions and biases, as suggested by the risk perception theory^19,20^. The preference for scientific literature among a quarter of respondents, particularly those with higher education, reflects the role of objective, evidence-based sources in reducing emotional and cognitive biases, supporting the principles of consumer choice theory^13,14^. The finding that social networks and personal contacts were the least common sources of information about healthy and safe food is notable. While social networks can be influential in shaping opinions and behaviors, particularly among younger demographics, their role in disseminating accurate information about food safety may be limited. Similarly, information obtained from friends, neighbors, and relatives may not always be based on scientific evidence, leading to potential misinformation or misconceptions.

The application of K-means clustering effectively segmented respondents into three distinct groups based on their perceptions of environmental, animal, and human health risks, awareness of GMO consumption and labeling, and views on pesticide usage in GMOs. The three clusters identified, “Highly Concerned,” “Moderately Concerned,” and “Critical but Uncertain,” highlighted varying levels of concern regarding the risks of GMOs. Our findings are consistent with previous research that shows a significant portion of the population holds neutral to negative views on GMOs, especially concerning potential health risks^65,66^. Participants in the “Highly Concerned” cluster, who make up 34.9% of the sample, were particularly sensitive to these health risks, associating GMOs with increased pesticide use and strongly opposing GMO consumption. This group aligns with the findings of Alam et al.^67^, which noted that many consumers perceive GMOs as posing health risks while recognizing their potential benefits for food production. The “Moderately Concerned” cluster, representing 37.9% of respondents, shares similar concerns but appears more indifferent to consumption habits, labeling, and pesticide use, indicating that while these respondents are aware of the potential dangers, they are less likely to act on or prioritize these concerns. The third cluster, “Critical but Uncertain,” which accounts for 27.2% of the respondents, demonstrates a critical stance on the environmental, animal, and human health risks associated with GMOs but remains uncertain about GMO consumption and strongly believes that GMOs are not labeled. This cluster is reflective of the findings in Čábelková et al.^68^, where while health risks dominate, environmental concerns may be less significant in influencing consumer acceptance. Across all clusters, pesticide use in GMO crops emerged as a recurrent concern. Thus, in order to improve public understanding and acceptance, communication strategies should be tailored to each consumer group. For the “Highly Concerned” cluster, addressing health fears with transparent, science-backed information is critical. The “Moderately Concerned” group may benefit from education on the economic and environmental advantages of GMOs, while the “Critical but Uncertain” cluster would respond best to clearer GMO labeling and regulatory transparency. Providing accessible, evidence-based messaging, may lead to GMO acceptance, particularly among those whose skepticism stems from uncertainty rather than strong opposition^66,67^.

## 5. Conclusions

This study provides valuable insights into how consumer perceptions of GMOs influence sustainable agricultural practices. Our findings reveal a significant knowledge gap and widespread skepticism among Montenegrin consumers, particularly regarding health risks, environmental impact, and regulatory transparency. Despite the growing integration of GMOs into global food supply chains, public understanding remains limited, raising the need for more effective communication strategies to bridge the gap between scientific knowledge and consumer perception.

Tailored educational programs should be developed for different consumer groups, addressing their specific concerns and misconceptions. Programs targeting older demographics could focus on debunking myths about health risks, while younger groups could benefit from clearer information on GMO benefits and the environmental impacts of agricultural practices. Strategies for information dissemination should prioritize digital platforms, given their growing influence as a primary source of information. Collaborating with trusted online influencers and scientists to create engaging, evidence-based content could help oppose misinformation. A simplified labeling system could empower consumers to make informed decisions and rebuild trust in the food supply chain. Government agencies and scientific institutions should also strengthen public outreach efforts to clarify GMO cultivation regulations in Montenegro and the EU, reducing uncertainty and fostering greater trust in regulatory institutions. In line with risk perception theory, public campaigns should address health concerns transparently while emphasizing the strict safety regulations governing GMO cultivation. By directly engaging with these concerns, skepticism can be reduced, and public trust strengthened.

Future research should explore the effectiveness of these communication strategies in diverse social and cultural contexts. Understanding how tailored messaging impacts different consumer groups will be critical to ensuring that policies are adapted to local needs. Finally, effort is needed to facilitate dialogue between consumers, regulatory authorities, and the scientific community. By addressing public concerns with empathy, transparency, and scientific rigor, policymakers and scientists can enhance public understanding of GMOs and support their role in sustainable agriculture. Achieving this balance between innovation and consumer trust may ensure that biotechnology contributes meaningfully to global food security and environmental sustainability.

## References

[1] Cortés AJ, Du H. Molecular genetics enhances plant breeding. Int J Mol Sci. 2023;24(12):997. doi: 10.3390/ijms24129977. 37373125 PMC10298300

[2] Husby J. Definitions of GMO/LMO and modern biotechnology. In: Traavick T Lim LC, editors. Biosafety first: holistic approaches to risk and uncertainty in genetic engineering and genetically modified organisms. Trondheim (NO): Tapir Academic Publishers; 2007. p. 8.

[3] Kumar NM, Muthukumaran C, Sharmila G, Gurunathan B. Genetically modified organisms and their impact on the enhancement of bioremediation. In: Varjani S, Agarwal A, Gnansounou E Gurunathan B, editors. Bioremediation: applications for environmental protection and management. Singapore: Springer; 2018. p. 67–14. doi: 10.1007/978-981-10-7485-1_4.

[4] Webb TL, Hong E. Gmo medicines and hospital pharmacy practice: a review. J Pharm Pract Res. 2021;51(3):203–10. doi: 10.1002/jppr.1742.

[5] Van Eenennaam AL. Gmos in animal agriculture: time to consider both costs and benefits in regulatory evaluations. J Anim Sci Biotechnol. 2013;4, (1):Article 37. doi: 10.1186/2049-1891-4-37. PMC4015968 24066781

[6] Saxena G, Kishor R, Saratale GD, Bharagava RN. Genetically modified organisms (GMOs) and their potential in environmental management: constraints, prospects, and challenges. In: Bharagava RN Saxena G, editors. Bioremediation of industrial waste for environmental safety. Singapore: Springer; 2020. p. 1–24. doi: 10.1007/978-981-13-3426-9_1.

[7] Uslu T. Advantages, risks and legal perspectives of GMOs in 2020s. Plant Biotechnol Rep. 2021;15(6):741–51. doi: 10.1007/s11816-021-00714-0.

[8] Lynas M, Adams J, Conrow J. Misinformation in the media: global coverage of GMOs 2019–2021. GM Crops Food Biotechnol Agri Food Chain. 2025;16(1):18–27. doi: 10.1080/21645698.2022.2140568. PMC11702960 36384421

[9] Van Eenennaam AL. Gene editing: from the general public perspective. J Anim Sci. 2024;102(Supplement_2):14. doi: 10.1093/jas/skae102.018.

[10] Jiang S, Fang W. Misinformation and disinformation in science: examining the social diffusion of rumours about GMOs. Cult Sci. 2019;2(4):327–40. doi: 10.1177/209660831900200407.

[11] Xu Q, Song Y, Yu N, Chen S. Are you passing along something true or false? Dissemination of social media messages about genetically modified organisms. Public Underst Sci. 2021;30(3):285–301. doi: 10.1177/0963662520966745. 33103588

[12] Ngo TTA, Phan TYN, Le TNT. Impacts of knowledge and trust on consumer perceptions and purchase intentions towards genetically modified foods. PLOS ONE. 2024;19(10):e0311257. doi: 10.1371/journal.pone.0311257. 39356695 PMC11446447

[13] Enke B, Graeber T. Cognitive uncertainty. Q J Econ. 2023;138(4):2021–67. doi: 10.1093/qje/qjad025.

[14] Siddiqui SA, Asif Z, Murid M, Fernando I, Adli DN, Blinov AV, Golik AB, Nugraha WS, Ibrahim SA, Jafari SM. Consumer social and psychological factors influencing the use of genetically modified foods—a review. Sustainability. 2022;14(23):15884. doi: 10.3390/su142315884.

[15] Liu X. The role of consumer behavior in shaping market demand and economic trends. Int J Educ Humanit. 2024;15(2):10–16. doi: 10.54097/skmxzd63.

[16] Goenka S, Thomas M. Moral foundations theory and consumer behavior. J Consum Phychol. 2024;34(3):536–40. doi: 10.1002/jcpy.1429.

[17] Cabelkova I, Sanova P, Hlavacek M, Broz D, Smutka L, Prochazka P. The moderating role of perceived health risks on the acceptance of genetically modified food. Front Public Health. 2024;11:1275287. doi: 10.3389/fpubh.2023.1275287. 38332939 PMC10851272

[18] Sikora D, Rzymski P. Public acceptance of GM foods: a global perspective (1999–2019). In: Singh P, Borthakur A, Singh AA, Kumar A, Singh KK, editors. Policy issues in genetically modified crops: a global perspective. Amsterdam, Netherlands: Elsevier; 2021. p. 293–315. doi: 10.1016/B978-0-12-820780-2.00013-3.

[19] Rembischevski P, Caldas ED. Risk perception related to food. Food Sci Technol. 2020;40(4):779–85. doi: 10.1590/fst.28219.

[20] Bawa AS, Anilakumar KR. Genetically modified foods: safety, risks and public concerns—a review. J Food Sci Technol. 2013;50(6):1035–46. doi: 10.1007/s13197-012-0899-1. 24426015 PMC3791249

[21] Malarkey T. Human health concerns with GM crops. Mutat Res Rev Mutat Res. 2003;544(2–3):217–21. doi: 10.1016/j.mrrev.2003.06.001. 14644323

[22] Spinks J, Nghiem S, Byrnes J. Risky business, healthy lives: how risk perception, risk preferences, and information influence consumer’s risky health choices. Eur J Health Econ. 2021;22(5):811–31. doi: 10.1007/s10198-021-01291-3. 33837875

[23] Chen MF. Consumer trust in food safety—a multidisciplinary approach and empirical evidence from Taiwan. Risk Anal Int J. 2008;28(6):1553–69. doi: 10.1111/j.1539-6924.2008.01115.x. 18793284

[24] Lam TK, Heales J, Hartley N, Hodkinson C. Consumer trust in food safety requires information transparency. Australas J Inf Syst. 2020;24:1–28. doi: 10.3127/ajis.v24i0.2219.

[25] Stoate C, Báldi A, Beja P, Boatman ND, Herzon I, van Doorn A, de Snoo GR, Rakosy L, Ramwell C. Ecological impacts of early 21st century agricultural change in Europe – a review. J Environ Manag. 2009;91(1):22–46. doi: 10.1016/j.jenvman.2009.07.005. 19717221

[26] Dinsmore DL, Zoellner BP, Parkinson MM, Rossi AM, Monk MJ, Vinnachi J. The effects of different types of text and individual differences on view complexity about genetically modified organisms. Int J Sci Educ. 2017;39(7):791–813. doi: 10.1080/09500693.2017.1298871.

[27] Basaran P, Kilic B, Soyyigit H, Sengun H. Public perceptions of GMOs in food in Turkey: a pilot survey. J Food Agri Environ. 2004;2(3):25–28. ISSN 1459-0255.

[28] Drobnjak R. Agriculture and entrepreneurship as a factor of sustainable development of Montenegro. In: Renko S Peštek A, editors. Green economy in the Western Balkans: towards a sustainable future. Bingley (UK): Emerald Publishing Limited; 2017. p. 395–419. doi: 10.1108/9781787144996.

[29] Jovović Z, Anđelković V, Pržulj N, Mandić D. Untapped genetic diversity of wild relatives for crop improvement. In: Salgotra R Zargar S, editors. Rediscovery of genetic and genomic resources for future food security. Singapore: Springer; 2020. p. 19–42.

[30] Djurovic G, Lajh D. Relationship with the European Union: Slovenia and Montenegro compared. Polit Central Eur. 2020;16(3):667–87. doi: 10.2478/pce-2020-0030.

[31] Chen JH, Gardner AK. Promoting inclusive environments through best practices in demographic survey design. Global Surg Educ J Assoc Surg Educ. 2022;1(1):Article 47. doi: 10.1007/s44186-022-00045-w.

[32] Meerza SIA, Dsouza A, Ahamed A, Mottaleb K. Risk propensity and acceptance of gene-edited and genetically modified food among US consumers: a comparison between plants and animal products. J Agric Appl Econ. 2024;56(4):575–96. doi: 10.1017/aae.2024.21.

[33] Kiran F, Sultan F, Nawaz MA. Acceptance of genetically modified organisms: a study based on consumption values, food attitude, and food technology neophobia. J Manag Sci. 2023;10(1):1–54.

[34] DeVellis RF, Thorpe CT. Scale development: theory and applications. 5th ed. Thousand Oaks (CA): SAGE Publications, Inc; 2022. ISBN: 978-1-5443-7934-0.

[35] Wu M-J, Zhao K, Fils-Aime F. Response rates of online surveys in published research: a meta-analysis. Comput Hum Behav Rep. 2022;7:100206. doi: 10.1016/j.chbr.2022.100206.

[36] Becker R. Gender and survey participation: an event history analysis of the gender effects of survey participation in a probability-based multi-wave panel study with a sequential mixed-mode design. Methods Data Anal. 2022;16(1):3–32. doi: 10.12758/mda.2021.08.

[37] King RS. Cluster analysis and data mining: an introduction. Dulles (VA): Mercury Learning and Information; 2015. ISBN: 978-1-938549-38-0.

[38] Marzban S, Allahyari MS, Damalas CA. Exploring farmers’ orientation towards multifunctional agriculture: insights from northern Iran. Land Use Policy. 2016;59:121–29. doi: 10.1016/j.landusepol.2016.08.020.

[39] Aerni P. Stakeholder attitudes towards the risks and benefits of genetically modified crops in South Africa. Environ Sci Policy. 2005;8(5):464–76. doi: 10.1016/j.envsci.2005.07.001.

[40] Bawa AS, Anilakumar KR. Genetically modified foods: safety, risks, and public concerns—a review. J Food Sci Technol. 2013;50(6):1035–46. 24426015 10.1007/s13197-012-0899-1 PMC3791249

[41] Motta R. Social disputes over GMOs: an overview. Sociol Compass. 2014;8(12):1360–76. doi: 10.1111/soc4.12229.

[42] Teferra TF. Should we still worry about the safety of GMO foods? Why and why not? A review. Food Sci Nutr. 2021;9(10):5364–75. doi: 10.1002/fsn3.2499. PMC8441473 34532037

[43] Lukošiutė I, Petrauskaitė-Senkevič L. Evaluation of Lithuanian consumers’ attitudes to genetically modified food. J Agribus Rural Devel. 2017;43(1):103–11. doi: 10.17306/J.JARD.2017.00336.

[44] Basaran P, Kilic B, Soyyigit H, Sengun H. Public perceptions of GMOs in food in Turkey: a pilot survey. J Food Agri Environ. 2004;2(3–4):25–28.

[45] Chen HY, Chern WS. Willingness to pay for GM foods: results from a public survey in the USA. In: Evenson RE, editor. Consumer acceptance of genetically modified foods. Wallingford (UK): CABI Publishing; 2004. p. 117–29. doi: 10.1079/9780851997476.0117.

[46] Monaco A. The role of heuristics and biases in the choice of risk triggers for novel foods and GMOs in the European Union. Eur J Risk Regul. 2024;16(Special Issue 1):217–27. doi: 10.1017/err.2024.48.

[47] Desaint N, Varbanova M. The use and value of polling to determine public opinion on GMOs in Europe: limitations and ways forward. GM Crops Food. 2013;4(3):183–94. doi: 10.4161/gmcr.26776. 24225741

[48] Zilberman D, Holland TG, Trilnick I. Agricultural GMOs—what we know and where scientists disagree. Sustainability. 2018;10(5):1514. doi: 10.3390/su10051514.

[49] De Vos CJ, Swanenburg M. Health effects of feeding genetically modified (GM) crops to livestock animals: a review. Food Chem Toxicol. 2018;117:3–12. doi: 10.1016/j.fct.2017.08.031. 28843598

[50] FAO/WHO. Guideline for the conduct of food safety assessment of foods derived from recombinant-DNA plants. Rome: Codex Alimentarius Commission; 2009.

[51] EFSA (European Food Safety Authority). Guidance for risk assessment of food and feed from genetically modified plants. EFSA J. 2011;9(5):2150. doi: 10.2903/j.efsa.2011.2150. PMC13177335 42148424

[52] Tsatsakis AM, Nawaz MA, Tutelyan VA, Golokhvast KS, Kalantzi O-I, Chung DH, Kang SJ, Coleman MD, Tyshko N, Yang SH, et al. Impact on environment, ecosystem, diversity and health from culturing and using GMOs as feed and food. Food Chem Toxicol. 2017;107(Part A):108–21. doi: 10.1016/j.fct.2017.06.033. 28645870

[53] Sendhil R, Nyika J, Yadav S, Mackolil J, Workie E, Ragupathy R, Ramasundaram P. Genetically modified foods: bibliometric analysis on consumer perception and preference. GM Crops Food. 2022;13(1):65. doi: 10.1080/21645698.2022.2038525. 35400312 PMC9009926

[54] Lynas M, Adams J, Conrow J. Misinformation in the media: global coverage of GMOs 2019-2021. GM Crops Food. 2022;16(1):18–27. 36384421 10.1080/21645698.2022.2140568 PMC11702960

[55] Pappalardo G, D’Amico M, Lusk JL. Comparing the views of the Italian general public and scientists on GMOs. Int J Food Sci Technol. 2021;56(7):3641–50. doi: 10.1111/ijfs.14993.

[56] Jurkiewicz A, Zagórski J, Bujak F, Lachowski S, Florek-Łuszczki M. Emotional attitudes of young people completing secondary schools towards genetic modification of organisms (GMO) and genetically modified foods (GMF). Ann Agric Environ Med. 2014;21(1):205–11. doi: 10.5604/1232-1966.1108599. 24738526

[57] Law on Genetically Modified Organisms. Off Gaz Mont 22/2008. 2008.

[58] Zheng Q, Wang HH. Do consumers view the genetically modified food labeling systems differently? “Contains GMO” versus “Non-GMO” labels. Chin Eco. 2021;54(6):376–88. doi: 10.1080/10971475.2021.1890356.

[59] Bruetschy C. The EU regulatory framework on genetically modified organisms (GMOs). Transgenic Res. 2019;28(Suppl 2):169–74. doi: 10.1007/s11248-019-00149-y. 31321701

[60] Madsen K, Sandøe P. Herbicide-tolerant crops and pesticide use. Pest Manag Sci. 2005;61(3):318–25. doi: 10.1002/ps.976. 15627240

[61] Bonny S. Global adoption of herbicide-tolerant GM crops: impacts and trends. Environ Manag. 2016;57(1):31–48. doi: 10.1007/s00267-015-0589-7. 26296738

[62] Hirasawa R, Yachi Y, Yoshizawa S, et al. Quality and accuracy of Internet information concerning a healthy diet. Int J Food Sci Nutr. 2013;64(8):1007–13. doi: 10.3109/09637486.2013.812620. 23863089

[63] Swire-Thompson B, Lazer D. Public health and online misinformation: challenges and recommendations. Annu Rev Public Health. 2020;41(1):433–51. doi: 10.1146/annurev-publhealth-040119-094127. 31874069

[64] Bouclaous C, Al Kamand A, Daher R, et al. Digital health literacy and online information-seeking behavior of Lebanese university students in the time of the COVID-19 pandemic and infodemic. Nordic J Digital Literacy. 2021;18(1):60–77. doi: 10.18261/njdl.18.1.6.

[65] Rosales A, Fernández-Ardèvol M, Gómez-León M, et al. Old age is also a time for change: trends in news intermediary preferences among internet users in Canada and Spain. Humanit Soc Sci Commun. 2024;11(1):455. doi: 10.1057/s41599-024-02940-7.

[66] Rampold S, Greig J, Gibson J, Nelson H. Gmo or gm no? Segmenting a consumer audience to examine their perceptions of genetically modified products. Adv Agric Devel. 2023;4(1):48–61. doi: 10.37433/aad.v4i1.269.

[67] Cábelková I, Sanová P, Hlaváček M, Brož D, Smutka L, Procházka P. The moderating role of perceived health risks on the acceptance of genetically modified food. Front Public Health. 2024;11:1275287. doi: 10.3389/fpubh.2023.1275287. 38332939 PMC10851272

[68] Alam F, Saha N, Islam M, Ahmed M, Haque M. Perception on environmental concern of pesticide use in relation to framers’ knowledge. J Environ Sci Nat Resour. 2022;13(1–2):94–99. doi: 10.3329/jesnr.v13i1-2.60696.

